# Lysophosphatidylcholine acyltransferase 4 is involved in chondrogenic differentiation of ATDC5 cells

**DOI:** 10.1038/s41598-017-16902-4

**Published:** 2017-12-01

**Authors:** Shirou Tabe, Hisako Hikiji, Wataru Ariyoshi, Tomomi Hashidate-Yoshida, Hideo Shindou, Takao Shimizu, Toshinori Okinaga, Yuji Seta, Kazuhiro Tominaga, Tatsuji Nishihara

**Affiliations:** 1 Division of Infections and Molecular Biology, Department of Health Promotion, Kyushu Dental University, Kitakyushu, Fukuoka, 803-8580 Japan; 2 Division of Oral and Maxillofacial Surgery, Department of Science of Physical Functions, Kyushu Dental University, Kitakyushu, Fukuoka, 803-8580 Japan; 3 School of Oral Health Sciences, Kyushu Dental University, Kitakyushu, Fukuoka, 803-8580 Japan; 4 Department of Lipid Signaling, Research Institute, National Center for Global Health and Medicine, Shinjuku-ku, Tokyo, 162-8655 Japan; 5 Agency for Medical Research and Development-Core Research for Evolutional Medical Science and Technology (AMED-CREST), AMED, Chiyoda-ku, Tokyo, 100-0004 Japan; 6 Department of Lipidomics, Graduate School of Medicine, The University of Tokyo, Bunkyo-ku, Tokyo, 113-0033 Japan; 7 Division of Anatomy, Department of Health Improvement, Kyushu Dental University, Kitakyushu, Fukuoka, 803-8580 Japan

## Abstract

Glycerophospholipids have important structural and functional roles in cells and are the main components of cellular membranes. Glycerophospholipids are formed via the *de novo* pathway (Kennedy pathway) and are subsequently matured in the remodeling pathway (Lands’ cycle). Lands’ cycle consists of two steps: deacylation of phospholipids by phospholipases A_2_ and reacylation of lysophospholipids by lysophospholipid acyltransferases (LPLATs). LPLATs play key roles in the maturation and maintenance of the fatty acid composition of biomembranes, and cell differentiation. We examined whether LPLATs are involved in chondrogenic differentiation of ATDC5 cells, which can differentiate into chondrocytes. Lysophosphatidylcholine acyltransferase 4 (LPCAT4) mRNA expression and LPCAT enzymatic activity towards 18:1-, 18:2-, 20:4-, and 22:6-CoA increased in the late stage of chondrogenic differentiation, when mineralization occurred. LPCAT4 knockdown decreased mRNA and protein levels of chondrogenic markers as well as Alcian blue staining intensity and alkaline phosphatase activity in ATDC5 cells. These results suggest that LPCAT4 plays important roles during the transition of chondrocytes into hypertrophic chondrocytes and/or a mineralized phenotype.

## Introduction

Glycerophospholipids have major functional and structural roles in cellular membranes. They provide precursors of lipid mediators, such as prostaglandins, leukotrienes, platelet-activating factor, and lysophospholipids^1,2^. There are different classes of glycerophospholipids: phosphatidic acid, phosphatidylcholine (PC), phosphatidylethanolamine, phosphatidylglycerol, phosphatidylinositol, phosphatidylserine, and cardiolipin^2,3^. The fatty acids in glycerophospholipids can widely vary between cell types and tissues. PC is the most abundant glycerophospholipid in mammalian cell membranes^4^. It plays a role in balancing the proportion of bilayer lipids that determine membrane intrinsic curvature^5^.

Glycerophospholipids are formed from glycerol-3-phosphate in the *de novo* pathway (Kennedy pathway^6^), and are subsequently remodeled by deacylation and reacylation (Lands’ cycle^7^). In the Land’s cycle, the concerted actions of phospholipases A_2_ and lysophospholipid acyltransferases (LPLATs) modify the fatty acid composition of glycerophospholipids^1,7,8^. LPLATs, which are important for maintaining an appropriate fatty acid composition of glycerophospholipids in cells, are widely distributed in tissues and show varying substrate preferences for acyl-CoAs and lysophospholipids^1,8^. LPLATs have been identified in the membrane-bound *O-*acyltransferase (MBOAT) and 1-acyl-glycerol-3-phosphate *O-*acyltransferase families^1,8^. Lysophosphatidylcholine acyltransferases (LPCATs), including LPCAT1–4, have LPLAT activities other than LPCAT activity. For example, LPCAT4 has lysophosphatidylethanolamine acyltransferase as well as LPCAT activity^8,9^. LPCAT uses lysophosphatidylcholine (LPC) as a substrate to generate PC^8,9^. LPC is involved in the pathogenesis of various lung disorders, including acute respiratory distress syndrome^10^. LPCAT4 mRNA is highly expressed in the epididymis, brain, testis, and ovary; however, the cellular functions of this enzyme remain unknown^8,9^.

Several LPLATs have important roles in organogenesis^9^. However, the role of LPLATs in chondrogenic differentiation has not yet been reported. We examined their expression to know the role of LPATs in chondrogenic differentiation in ATDC5 cells. The clonal chondrogenic cell line ATDC5 was isolated from the differentiating teratocarcinoma stem cell line, AT805^11^, and is widely utilized to study chondrocyte growth and differentiation *in vitro* ^12^. Based on the results, further analyses were focused on LPCAT4.

LPCAT4 mRNA expression and LPCAT enzymatic activity increased in the late stage of chondrogenic differentiation when mineralization occurred. Knockdown of LPCAT4 suppressed the mRNA levels of chondrogenic differentiation markers, Col10, alkaline phosphatase (ALP), aggrecan, and transforming growth factor-β (TGF-β) and protein expression of Col10. The knockdown of LPCAT4 also suppressed Alcian blue staining intensity and ALP activity. Furthermore, knockdown of LPCAT4 suppressed mRNA levels of bone morphogenetic proteins (BMPs), molecules concerning cell signaling pathways known to regulate chondrogenic differentiation of ATDC5 cells. These results suggest that LPCAT4 plays a role as chondrocytes transition into hypertrophic chondrocytes and/or a mineralized phenotype.

## Results

### Differentiation of ATDC5 into chondrogenic cells

**Figure 1:**
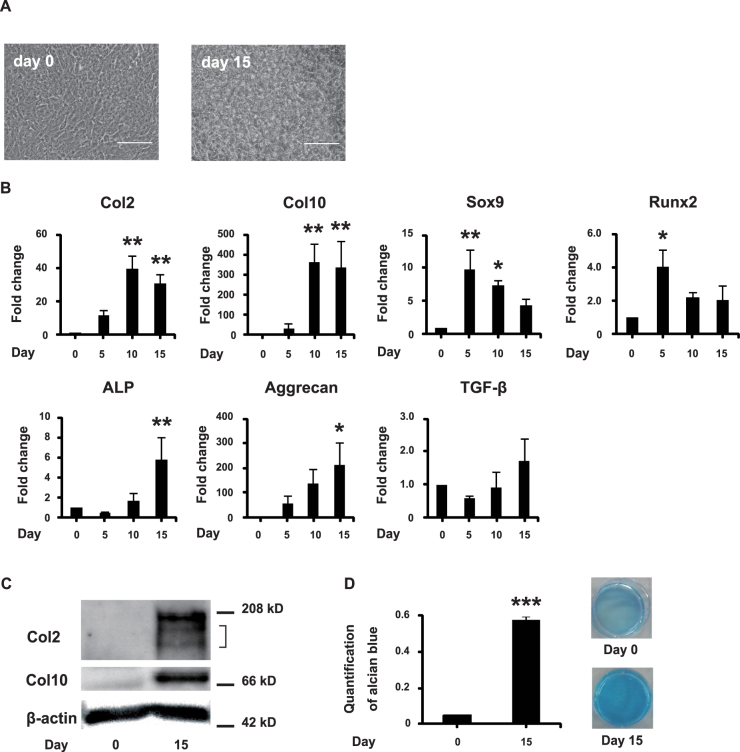
ATDC5 cells differentiate into chondrogenic cells. ATDC5 cells were seed in 6-well tissue culture plates and cultured in α-MEM with 50 ng/ml ascorbic acid and 1% ITS. (**A**) Representative images of phase-contrast micrography of ATDC5 cells. Scale bar is 100 μm. (**B**) mRNA levels of the chondrogenic differentiation markers, Col2, Col10, Sox9, Runx2, ALP, aggrecan, and TGF-β. The fold increase in mRNA expression was calculated relative to day 0 (day 0 = 1) and normalized to GAPDH. Error bars represent mean ± S.D., n = 3. *p < 0.05; **p < 0.01, compared with day 0, post-hoc test (Bonferroni’s correction) after one-way ANOVA. (**C**) Protein expression of chondrogenic differentiation markers, Col2 and Col10, and of the internal control, β-actin. (**D**) Alcian blue staining was quantified. Alcian blue-stained cultures were extracted with 6 M guanidine-HCl. The absorbance of the digests was measured at 630 nm. Error bars represent mean ± S.D., n = 3. ***p < 0.001, Student’s *t*-test was used to compare day 0 and day 15. The left panel shows the quantification of Alcian blue staining. The right panel shows representative images of ATDC5 cells stained with Alcian blue on days 0 and 15.

ATDC5 cells were cultured in α-MEM with ascorbic acid and insulin-transferrin-selenium (ITS). Nodule formation was observed during chondrogenic differentiation (Fig. 1A). Col2 and Col10 mRNA levels increased, and peaked at day 10 (Fig. 1B). Sox9 and Runx2 mRNA expression decreased after peaking at day 5 (Fig. 1B). ALP, aggrecan, and TGF-β are chondrogenic differentiation markers. ALP expression is indicative of mineralization that occurs subsequently^13^. mRNA expression of these markers, except for TGF-β, increased during chondrogenic differentiation (Fig. 1B). The protein expression of Col2 and Col10 increased on day 15 (Fig. 1C). Alcian blue staining, used to visualize proteoglycan synthesis, also increased on day 15 (Fig. 1D). These results indicated that ATDC5 cells effectively differentiated into chondrogenic cells.

### LPCAT4 mRNA expression and LPCAT enzymatic activity increase during chondrogenic differentiation of ATDC5 cells

**Figure 2:**
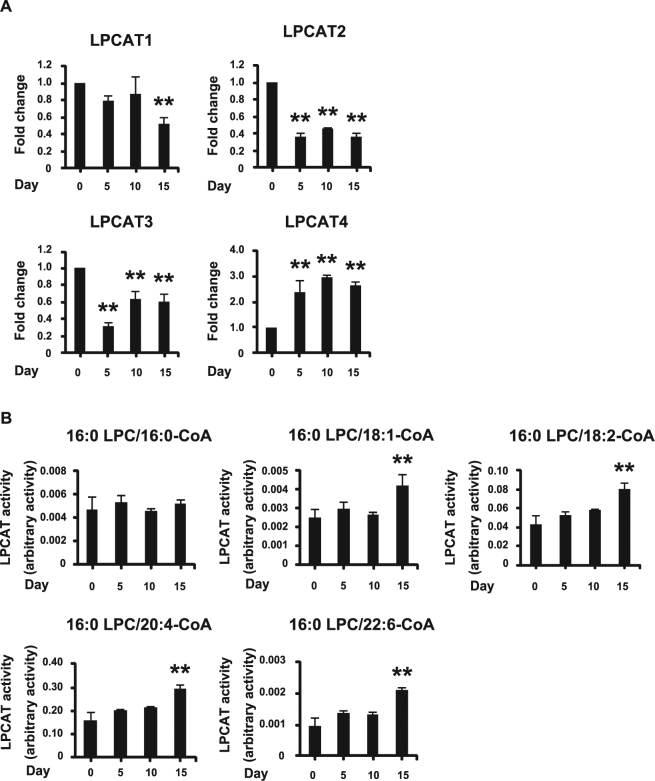
LPCAT4 mRNA expression and LPCAT enzymatic activity increase during chondrogenic differentiation of ATDC5 cells. (**A**) mRNA levels of LPCATs in ATDC5 cells cultured in α-MEM with 50 ng/ml ascorbic acid and 1% ITS. The fold increase in mRNA expression was calculated relative to day 0 (day 0 = 1) and normalized to GAPDH. Error bars represent mean ± S.D., n = 3. **p < 0.01, compared with day 0, post-hoc test (Bonferroni’s correction) after one-way ANOVA. (**B**) LPCAT enzymatic activity in ATDC5 cells measured with 16:0-, 18:1-, 18:2-, 20:4-, and 22:6-CoA as donors. Error bars represent mean ± S.D., n = 3. **p < 0.01, compared with day 0, post-hoc test (Bonferroni’s correction) after two way ANOVA.

LPCAT4 mRNA expression increased during chondrogenic differentiation (Fig. 2A), while mRNA levels of LPCAT1, LPCAT2, and LPCAT3 decreased (Fig. 2A). LPCAT enzymatic activity in the presence of 18:1-, 18:2-, 20:4-, and 22:6-CoA increased on day 15, while it remained unchanged in the presence of 16:0-CoA (Fig. 2B). These results implicate a preference of LPCAT4 for 18:1-CoA during chondrogenic differentiation. However, LPCAT4 does not prefer 18:2-, 20:4-, or 22:6-CoA, suggesting the existence of unknown enzymatic activities with preference for these fatty acids in ATDC5 cells. Analysis of the fatty acid composition of whole cell lysates by liquid chromatography-selected reaction monitoring/mass spectrometry (LC-SRM-MS) revealed that the percentage of PC was not detected during chondrogenic differentiation of ATDC5 cells (Supplementary Table S1).

### LPCAT4 mRNA expression increases during chondrogenic differentiation of C3H10T1/2 cells

**Figure 3:**
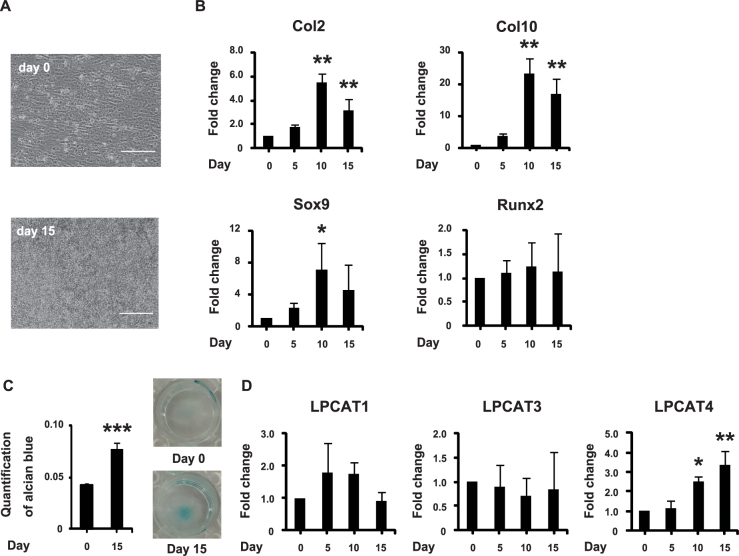
LPCAT4 mRNA expression increases during chondrogenic differentiation of C3H10T11/2 pre-chondrogenic cells. Drops of highly concentrated C3H10T1/2 cells were plated on tissue culture plates and cultured in DMEM/F-12 containing 100 ng/ml human recombinant BMP2. (**A**) Representative images of phase-contrast micrography of C3H10T1/2 cells. Scale bar is 100 μm. (**B**) mRNA levels of the chondrogenic differentiation markers, Col2, Col10, Sox9, and Runx2. The fold increase in mRNA expression was calculated relative to day 0 (day 0 = 1) and normalized to GAPDH. Error bars represent mean ± S.D., n = 3. *p < 0.05; **p < 0.01, compared with day 0, post-hoc test (Bonferroni’s correction) after two-way ANOVA. (**C**) Alcian blue staining was quantified. Error bars represent mean ± S.D., n = 3. ***p < 0.01, Student’s *t*-test was used to compare day 0 and day 15. The left panel shows the quantification of Alcian blue staining. The right panel shows representative images of C3H10T1/2 cells stained with Alcian blue on days 0 and 15. (**D**) mRNA levels of LPCAT1, LPCAT3, and LPCAT4. The fold increase in mRNA expression was calculated relative to day 0 (day 0 = 1) and normalized to GAPDH. The error bars represent mean ± S.D., n = 3. *p < 0.05; **p < 0.01, compared with day 0, post-hoc test (Bonferroni’s correction) after one-way ANOVA.

C3H10T1/2 cells, a pluripotent embryonic murine mesenchymal cell line, undergo chondrogenic differentiation in the presence of BMP2 in high-density micromass culture^14,15^. Nodule formation was observed during chondrogenic differentiation of C3H10T1/2 cells (Fig. 3A). mRNA expression of Col2, Col10, and Sox9 increased, peaking on day 10 (Fig. 3B), while Runx2 mRNA did not change. Alcian blue staining of C3H10T1/2 cells increased on day 15 (Fig. 3C). LPCAT4 mRNA expression increased in a time-dependent manner (Fig. 3D). However, LPCAT 1 and LPCAT3 mRNA levels remained unchanged, and that of LPCAT2 was not detected.

To examine whether LPCAT4 is expressed in chondrocytes, we conducted *in situ* hybridization of mouse embryos using LPCAT4 probes, as well as probes for the chondrogenic markers, Col2 and Col10. Col10 is highly expressed in the hypertrophic zone of cartilage^13^. The results showed that LPCAT4 mRNA expression is stronger in the hypertrophic than in the prehypertrophic zone of cartilage (Supplementary Fig. S1).

### LPCAT4 knockdown inhibits hypertrophy/mineralization after a chondrogenic phenotype has been attained in ATDC5 cells

**Figure 4:**
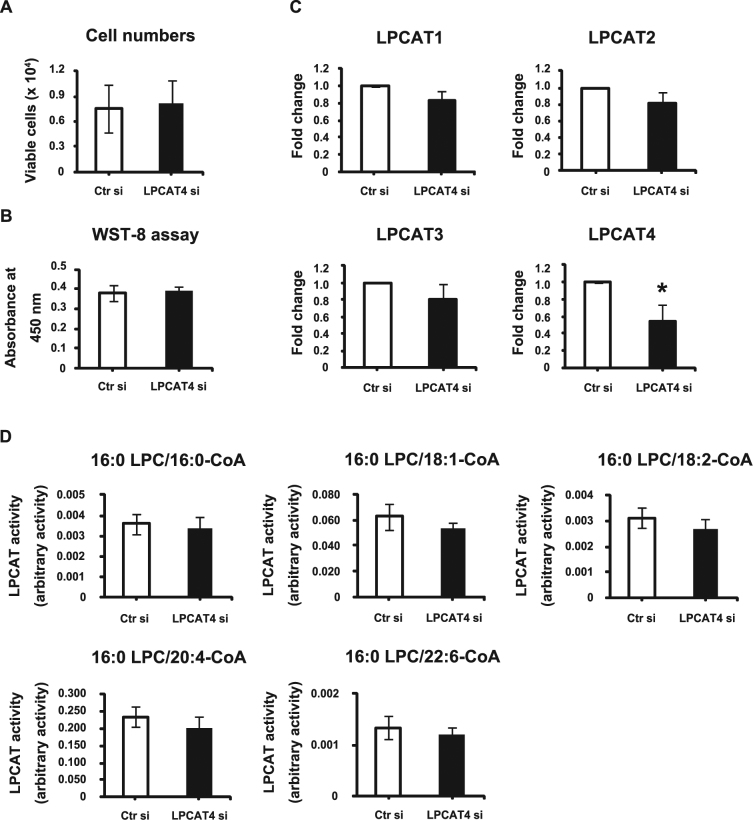
Effects of LPCAT4 knockdown on LPCAT enzymatic activity. ATDC5 cells were transfected with control siRNA (Ctr si) or LPCAT4 siRNA (LPCAT si) for 24 h. (**A**) Cell viability was quantified using trypan blue dye exclusion test 24 h after transfection. Cells were counted in 3 wells, and the mean values from 3 wells are presented. Error bars represent mean ± S.D., n = 3. Student’s *t*-test was used to compare Ctr si and LPCAT4 si. (**B**) Cell viability was determined by WST-8 assay 24 h after transfection. Error bars represent mean ± S.D., n = 3. Student’s *t*-test was used to compare Ctr si and LPCAT4 si. (**C**) Total RNA was extracted from ATDC5 cells on day 15 after transfection. The fold increase in mRNA expression was calculated relative to cells transfected with Ctr si (Ctr si = 1) and normalized to GAPDH. Error bars represent mean ± S.D., n = 3. *p < 0.05, Student’s *t*-test was used to compare Ctr si and LPCAT4 si. (**D**) LPCAT enzymatic activity in ATDC5 cells measured with 16:0-, 18:1-, 18:2-, 20:4-, and 22:6-CoA as donors on day 15 after transfection. Error bars represent mean ± S.D., n = 3. Student’s *t-*test was used to compare Ctr si and LPCAT4 si.

To confirm whether LPCAT4 functions in chondrogenic differentiation, we silenced its expression in ATDC5 cells. To this end, ATDC5 cells were transfected with LPCAT4 siRNA or a scramble siRNA, used as a negative control. Control siRNA, used as a negative control, consisted of a scrambled sequence that would not affect any cellular message and function. LPCAT4 siRNA-transfected cells maintained viability 24 h after transfection (Fig. 4A,B). The transfected ATDC5 cells were cultured in α-MEM with ascorbic acid and ITS to induce chondrogenic differentiation. LPCAT4 siRNA transfection specifically suppressed mRNA expression of LPCAT4, without affecting LPCAT1–3 transcript levels, on day 15 after transfection (Fig. 4C). In control siRNA-transfected cells, LPCAT4 transcripts increased during chondrogenic differentiation (data not shown). However, knockdown of LPCAT4 did not change LPCAT enzymatic activity (Fig. 4D) and the percentage of PC species (Supplementary Table S2).

**Figure 5:**
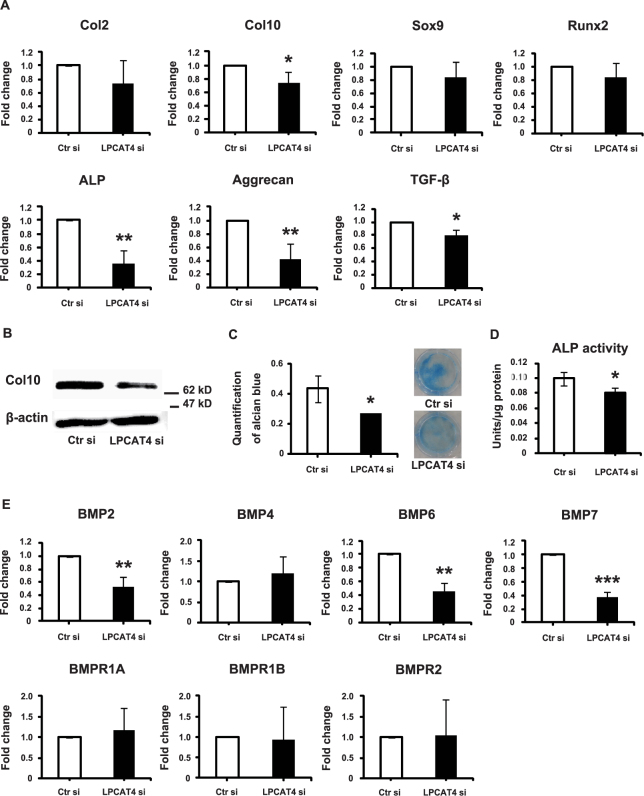
Effect of LPCAT4 knockdown on chondrogenic differentiation. ATDC5 cells were transfected with control siRNA (Ctr si) or LPCAT4 siRNA (LPCAT si) for 24 h. (**A**) Total RNA was extracted from ATDC5 cells on day 15 after transfection. mRNA levels of chondrogenic differentiation markers, Col2, Col10, Sox9, Runx2, ALP, aggrecan, and TGF-β. The fold increase was calculated relative to cells transfected with Ctr si (Ctr si = 1) and normalized to GAPDH. Error bars represent mean ± S.D., n = 3. *p < 0.05; **p < 0.01, Student’s *t*-test was used to compare Ctr si and LPCAT4 si. (**B**) Protein was extracted from ATDC5 cells on day 15 after transfection. Protein expression of the chondrogenic differentiation marker, Col10, and of the internal control, β-actin. (**C**) Alcian blue staining was quantified. Error bars represent mean ± S.D., n = 3. *p < 0.05, Student’s *t*-test was used to compare Ctr si and LPCAT4 si. The left panel shows the quantification of Alcian blue staining. The right panel shows representative images of ATDC5 cells transfected with Ctr si or LPCAT4 si and stained with Alcian blue on day 15 after transfection. (**D**) ALP activity in ATDC5 cells on day 15 after transfection. The fold increase was calculated relative to cells transfected with Ctr si (Ctr si = 1) and normalized to GAPDH. Error bars represent mean ± S.D., n = 3. *p < 0.05, Student’s *t*-test was used to compare Ctr si and LPCAT4 si. (**E**) Total RNA was extracted from ATDC5 cells on day 15 after transfection. mRNA levels of BMPs and BMPRs. The fold increase was calculated relative to cells transfected with Ctr si (Ctr si = 1) and normalized to GAPDH. Error bars represent mean ± S.D., n = 3. *p < 0.05, **p < 0.01, ***p < 0.001, Student’s *t-*test was used to compare Ctr si and LPCAT4 si.

Knockdown of LPCAT4 suppressed the mRNA expression of the chondrogenic differentiation markers, Col10, ALP, aggrecan, and TGF-β (Fig. 5A) and protein expression of Col10 (Fig. 5B) on day 15 after transfection. The expression of Col2, Sox9, and Runx2 did not change. Additionally, Alcian blue staining intensity and ALP activity were lower in LPCAT4 knockdown than in control cells on day 15 after transfection (Fig. 5C,D). These results suggested that LPCAT4 facilitates mineralization after the chondrogenic phenotype has been attained. ATDC5 cells express several BMPs during chondrogenic differentiation^16^. On day 15 after transfection, the knockdown of LPCAT4 suppressed the mRNA expression of BMP2, BMP6, and BMP7 during chondrogenic differentiation of ATDC5 cells (Fig. 5E).

## Discussion

In this study, we found that LPCAT4 expression increased during the chondrogenic differentiation of ATDC5 cells as well as that of C3H10T1/2 cells. In mouse embryos, the expression of LPCAT4 mRNA was stronger in the hypertrophic than in the prehypertrophic zone of cartilage. These results suggest that LPCAT4 is involved in hypertrophy/mineralization after attainment of the chondrogenic phenotype.

LPCAT4 converts LPC to PC, with preference for 18:1-CoA^8,9^. 16:0–18:1 PC levels were reportedly increased in Chinese hamster ovary K1 cells stably overexpressing LPCAT4^17^. 18:1 acyl chains may contribute to lipid-packing defects, which are loosely packed sites in the curved membrane produced by cone-shaped glycerophospholipids having small polar heads, and/or bulky acyl chains, including 18:1^9,18^. The regulation of cone-shaped glycerophospholipid biosynthesis by LPCAT4 exhibiting LPCAT enzymatic activity, may affect vesicle trafficking, membrane fusion, endocytosis, and exocytosis by introducing local lipid-packing defects in curved membranes^9^. Extracellular matrix (ECM) is composed of proteoglycans, such as aggrecan and Col2, and other molecular components^19,20^. ECM–chondrocyte interactions regulate cell growth, differentiation, and morphogenesis^19,20^. Therefore, LPCAT4 may participate in the morphological change of chondrocytes, including hypertrophy.

Although 18:1-CoA is the preferred substrate of LPCAT4^8,9^, in this study, LPCAT enzymatic activity towards 18:2-, 20:4-, and 22:6-CoA also increased during chondrogenic differentiation of ATDC5 cells, which may imply increased activity of other LPLATs, such as LPCAT1–3. LPCAT2 and 3, but not LPCAT1, are known to prefer 18:2- and/or 20:4-CoA^8,9^. However, LPCAT2/3 mRNA expression decreased during chondrogenic differentiation. LPLATs preferring 22:6-CoA have not been identified to date. Thus, the molecular mechanisms behind the changes in LPCAT activity towards 18:2-, 20:4-, and 22:6-CoA remain unresolved, and may involve hitherto unknown enzymes. At least, on the condition that the mRNA expression of LPCAT4 increases, the enzymatic activity for 18:1-CoA increases. Consistent with our previous finding that LPCAT4 prefers 18:1-CoA^8,9^, the enzymatic activity toward 18:1-CoA increased concomitantly with the upregulation of LPCAT4 mRNA expression.

Although LPCAT4 mRNA expression and LPCAT enzymatic activity increased during chondrogenic differentiation in ATDC5 cells, the fatty acid composition of PC species was unchanged by LC-SRM-MS analysis (Supplemental Tables S1). It has been reported that arachidonate enrichment by LPCAT3 produces a pool of triacylglycerol in local membranes, which is required for normal directionality of triacylglycerol transfer and lipoprotein assembly in the liver and enterocytes^21,22^. A unique component of phospholipids, 1-oleoyl-2-palmitoyl PC, localized at the protrusion tip in nerve growth factor-treated PC12 cells during neuronal differentiation^23^. We previously found that LPCAT4 mainly localized to the ER when overexpressed in Chinese hamster ovary K1 cells^24^. It is possible that specific changes in glycerophospholipid fatty acids in the local biomembrane affect cellular morphology and function in hypertrophy/mineralization of ATDC5 cells. However, we observed no changes in the fatty acid composition of PC species in phospholipids from whole cell lysates by LC-SRM-MS analysis. The local enrichment of particular fatty acid species will have to be determined in future studies using imaging MS with a higher resolution or other strategies.

Upregulation of LPCAT4 mRNA was observed from 5 days of differentiation, while LPCAT activity was enhanced only after 15 days. This 10-day delayed increase in LPCAT activity as compared to gene expression, rather than synchronized mRNA expression and enzymatic activity increase, led us to speculate that not all transcribed LPCAT4 is translated into an active enzyme. Alternatively, the sensitivity of our assay to detect enzymatic activity might have been insufficient; while LPCAT4 enzymatic activity was clearly detected in normal ATDC5 cells, no change in activity could be detected in LPCAT4 knockdown ATDC5 cells.

Knockdown of LPCAT4 did not change LPCAT enzymatic activity towards 16:0-, 18:1-, 18:2-, 20:4-, and 22:6-CoA in ATDC5 cells during chondrogenic differentiation measured, which may be explained by the *in vitro* experimental conditions that obviously differ from the cellular environment *in vivo*, including protein content and the amount of substrate, or by insufficient assay sensitivity as mentioned above. Alternatively, LPCAT4 might have a hitherto unknown function other than LPCAT activity using LPC as a substrate to produce PC. For example, LPCAT4 may directly bind other proteins such as transcription factors or it may function as a scaffold protein.

Knockdown of LPCAT4 decreased the mRNA expression of molecules related to chondrogenic differentiation other than Col2, Sox9, and Runx2. Aggrecan is the major proteoglycan component in the ECM of articular cartilage^25^. TGF-β, which is known as an inducer of chondrogenic differentiation, mediates mRNA expression of aggrecan^26–28^. Although the relationship among LPCAT4, aggrecan, and TGF-β in chondrocytes remains unknown, these molecules may collaborate. Immature chondrocytes from the prechondrogenic to the proliferative stage express Col2^29^, and mature chondrocytes from the proliferative/prehypertrophic to the hypertrophic stage express Col10^30^. Chondrocyte hypertrophic differentiation is characterized by the expression of hypertrophic markers such as Col10 *in vitro* ^31^. Sox9 is expressed in immature chondrocytes^30,31^. Runx2 is a transcriptional factor that promotes prehypertrophic cells to enter hypertrophy^30,31^, suggesting that Runx2 plays a role in prehypertrophic cells before mineralization. Knockdown of LPCAT4 decreased the mRNA expression of Col10, but not of Col2, Sox9, and Runx2. Additionally, LPCAT4 knockdown decreased the protein expression of Col10. *In situ* hybridization showed that LPCAT4 mRNA expression in the hypertrophic zone of cartilage was stronger than that in the prehypertrophic zone. Furthermore, Alcian blue staining intensity was decreased in LPCAT4 knockdown ATDC5 cells. Together, these results suggest that knockdown of LPCAT4 inhibits hypertrophy/mineralization after attainment of the chondrogenic phenotype. In C3H10T1/2 cells, Col2 and Col10 expression actually started to diminish after day 10, while LPCAT4 expression still tended to increase at day 15. This finding suggests that LPCAT4 may have different roles in ATDC5 and C3H10T1/2 cells up to the point of hypertrophy. Further, the data seem to suggest that LPCAT4 might function in the progression into a hypertrophic phenotype and afterwards.

ALP is one of the functional markers of prehypertrophic chondrocytes^32,33^ and contributes to the mineralization of cartilage ECM^34^. Knockdown of LPCAT4 decreased ALP activity on day 15, suggesting that LPCAT4 is involved in the late stage of chondrogenic differentiation, when mineralization occurs.

BMP signaling regulates the chondrogenic differentiation of ATDC5 cells^16,35,36^. ATDC5 cells reportedly express BMP2, BMP4, BMP6, and BMP7^16^. Among these, BMP6 and BMP7 are expressed in mature chondrocytes^37–39^. BMP2 induces early-phase chondrogenic differentiation of ATDC5 cells as well as late-phase differentiation (hypertrophic chondrocytes and matrix mineralization)^35^. BMP4 and BMPR2 are expressed in all stages of chondrogenic differentiation^16^. Overexpression of BMPR1A and BMPR1B induces chondrogenesis in ATDC5 cells^40^. LPCAT4 knockdown decreased the mRNA expression of several BMPs, including BMP2, BMP6, and BMP7, in ATDC5 cells, suggesting that LPCAT4 is related to BMP expression.

In conclusion, we showed that LPCAT4 regulates hypertrophy/mineralization after establishment of the chondrogenic phenotype in ATDC5 cells. LPCAT4 may have potential as a therapeutic target for articular cartilage disorders.

## Methods

### Reagents

Horseradish peroxidase (HRP)-conjugated anti-mouse IgG and LPCAT4 siRNA (ON-TARGET plus mouse MBOAT2: L-063482-01) were purchased from GE Healthcare (Little Chalfont, UK). Anti-Col2 polyclonal antibody, anti-Col10 polyclonal antibody, control siRNA (SC: 37007), and HRP-conjugated donkey anti-goat IgG were purchased from Santa Cruz Biotechnology (Santa Cruz, CA, USA). Anti-β-actin monoclonal antibody, bovine serum albumin (BSA), and 0.25% trypsin-EDTA were purchased from Sigma-Aldrich (St. Louis, MO, USA).

### Cell culture and chondrogenic differentiation

ATDC5 cells (Riken Bio Resource Center Cell Bank, Ibaragi, Japan) were maintained in Dulbecco’s Modified Eagle Medium: Nutrient Mixture F-12 (DMEM/ F-12; Thermo Fisher Scientific Life Sciences, Waltham, MA, USA) supplemented with 5% fetal bovine serum (Sigma-Aldrich), 100 U/ml penicillin G potassium salt (Nacalai Tesque, Kyoto, Japan) and 100 μg/ml streptomycin (Wako, Osaka, Japan) at 37 °C in an atmosphere of 95% air and 5% CO_2_ at 37 °C and 5% CO_2_.

C3H10T1/2 cells (Riken Bio Resource Center Cell Bank) were maintained in DMEM/F-12 supplemented with 10% fetal bovine serum, 100 U/ml penicillin G potassium salt and 100 μg/ml streptomycin at 37 °C in an atmosphere of 95% air and 5% CO_2_ at 37 °C and 5% CO_2_.

To induce chondrogenic differentiation, ATDC5 cells were seeded in 6-well tissue culture plates at a density 3.0 × 10^5^ cells/well and cultured in the differentiation medium containing α-MEM (Thermo Fisher Scientific Life Sciences) with 50 ng/ml ascorbic acid (Wako) and 1% insulin-transferrin-selenium (ITS: 1 mg/ml insulin, 0.55 mg/ml transferrin, 0.67 μg/ml selenium; Thermo Fisher Scientific Life Sciences). The medium was changed every 2 days.

To induce chondrogenic differentiation, C3H10T1/2 cells were trypsinized and resuspended at a concentration of 2.0 × 10^7^ cells/ml. Ten-microliter drops of cell suspension were seeded in 24-well tissue culture plates and allowed to adhere for 2–3 h, and then 1 ml of DMEM/F-12 containing 100 ng/ml human recombinant BMP2 (R&D Systems, Minneapolis, MN, USA) was added to the cultures. The medium was changed every 3 days.

### Quantitative real-time RT-PCR

Total RNA was extracted from the cells using the Cica geneus RNA Prep Kit (Kanto Chemical, Tokyo, Japan) according to the manufacturer’s instructions. RNA was transcribed with ReverTra Ace qPCR RT Kit (Toyobo, Osaka, Japan), and cDNA was amplified using a Mastercycler gradient (Eppendorf, Hamburg, Germany). Quantitative PCRs were performed with Step One Real-Time System (Thermo Fisher Scientific Life Sciences) or Light Cycler System (Roche, Tokyo, Japan) using FAST SYBR Green Master Mix (Thermo Fisher Scientific Life Sciences) or FastStart Essential DNA Green Master (Roche), as appropriate. Target gene mRNA levels were normalized to that of glyceraldehyde-3-phosphate dehydrogenase (GAPDH). The primers used were: LPCAT1, 5′-GTGCACGAGCTGCGACT-3′ (forward) and 5′-GCTGCTCTGGCTCCTTATCA-3′ (reverse); LPCAT2, 5′-GTCCAGCAGACTACGATCAGTG-3′ (forward) and 5′-CTTATTGGATGGGTCAGCTTTTC-3′ (reverse); LPCAT3, 5′-TCAGGATACCTGATTTGCTTCCA-3′ (forward) and 5′-GGATGGGTCTGTTGCACCAAGTAG-3′ (reverse); LPCAT4, 5′-TTCGGTTTCAGAGGATACGACAA-3′ (forward) and 5′-AATGTCTGGATTGTCGGACTGAA-3′ (reverse); Col2, 5′-AAGTCACTGAACAACCAGATTGAGA-3′ (forward) and 5′-AAGTGCGAGCAGGGTTCTTG-3′ (reverse); Col10, 5′-TGCAATCATGGAGCTCACAGA-3′ (forward) and 5′-CAGAGGAGTAGAGGCCGTTTGA-3′ (reverse); Sox9, 5′-ACCCACCACTCCCAAAACC-3′ (forward) and 5′-CGCCCCTCTCGCTTCAG-3′ (reverse); Runx2, 5′-GCCGAGCTCCGAAATGC-3′ (forward) and 5′-AGATCGTTGAACCTGGCTACTTG-3′ (reverse); ALP, 5′-GGTATGGGCGTCTCCACAGT-3′ (forward) and 5′-GCCCGTGTTGTGGTGTAGCT-3′ (reverse); aggrecan, 5′-ACGCCCCGGGAAGGT-3′ (forward) and 5′-CCGGATTCCGTAGGTTCTCA-3′ (reverse); TGF-β, 5′-GGTGGTATACTGAGACACCTTG-3′ (forward) and 5′-CCCAAGGAAAGGTAGGTGATAG-3′ (reverse); BMP2, 5′-ACACAGCTGGTCACAGATAAG-3′ (forward) and 5′-CTTCCGCTGTTTGTGTTTGG-3′ (reverse); BMP4, 5′-AACGTAGTCCCAAGCATCAC-3′ (forward) and 5′-CGTCACTGAAGTCCACGTATAG-3′ (reverse); BMP6, 5′-CCAACTACTGTGATGGAGAGTG-3′ (forward) and 5′-GGACGTACTCGGGATTCATAAG-3′ (reverse); BMP7, 5′-AGAGGTGGGATGTTGGTTATG-3′ (forward) and 5′-CCAGTTTAACCCTCTGCATTTG-3′ (reverse); BMPR1A, 5′-GAGTGGATCTGGATTGCCTTTA-3′ (forward) and 5′-CGCCATTTACCCATCCATACT-3′ (reverse); BMPR1B, 5′-CTCCTGCCCGTTCTTTATACTC-3′ (forward) and 5′-GACCACACACCAAACGAAATG-3′ (reverse); BMPR2, 5′-GCAATCTCCCACCGAGATTTA-3′ (forward) and 5′-ACCAGCCGATTTCCAGTTAG-3′ (reverse); and GAPDH, 5′-TGACAATGAATACGGCTACAGCA-3′ (forward) and 5′-CTCCTGTTATTATGGGGGTCTGG-3′ (reverse).

### Western blot analysis

Total protein was extracted using Cell Lysis Buffer (Cell Signaling Technology, Beverly, MA, USA) containing a proteinase inhibitor cocktail (Thermo Fisher Scientific Life Sciences). Protein concentration was measured with the DC protein assay kit (Bio-Rad, Hercules, CA, USA) using BSA as a standard. Equivalent amounts of total protein were separated on 7.5% polyacrylamide gels. Proteins were transferred onto polyvinylidene difluoride membranes (Millipore, Bedford, MA, USA) and blocked with Blocking One (Nacalai Tesque) for 1 h at room temperature (RT). Membranes were incubated overnight at 4 °C with diluted primary antibody: anti-Col2 (1000:1), anti-Col10 (1000:1), and anti-β-actin (5000:1), followed by HRP-conjugated anti-mouse, anti-rabbit or donkey anti-goat IgG for 1 h at RT. Chemiluminescence was performed using ECL reagent (GE Healthcare) and detected digitally with a Molecular Imager Chasidic XRS Plus system (Bio-Rad).

### Alcian blue staining

ATDC5 and C3H10T1/2 cells were fixed with 4% paraformaldehyde (Nacalai Tesque) for 10 min at 4 °C, and stained with Alcian blue stain solution (pH 1; Muto Chemical, Tokyo, Japan) overnight at RT. For quantification, the stained cultures were extracted with 6 M guanidine-HCl (Wako) overnight at RT. The absorbance of the digests was measured at 630 nm with Multiskan JX (Thermo Fisher Scientific Life Sciences).

### LPCAT enzymatic activity assay

ATDC5 cells were scraped into 1 ml of ice-cold buffer containing 20 mM Tris-HCl (pH 7.4), 300 mM sucrose, and protease inhibitor cocktail, Complete (Roche). Cells were sonicated on ice two times each for 30 s using a probe sonicator (Ohtaka Works, Tokyo, Japan). The protein solutions were collected from supernatants centrifuged at 800 × *g* for 10 min. Protein concentration was measured by Bio-Rad Protein Assay (Bio-Rad). LPCAT enzymatic assay was performed as previously described^17,21^. Briefly, proteins (0.1 µg) were mixed with 25 µM deuterium-labeled 16:0 LPC and 1 µM each of 16:0-, 18:1-, 18:2-, 20:4-, and 22:6-CoA (Avanti Polar Lipids, Pelhan, AL) at 37 °C for 10 min. Reaction was stopped by the addition of 300 µl chloroform/methanol (1:2; Wako). Reaction mixtures contained 150 mM Tris-HCl (pH 7.4), 1.5 mM EDTA, 2 mM CaCl_2_ (Wako), 0.015% Tween-20, and 150 mM sucrose. An internal standard, dilauryl-PC, was added and lipids were extracted by the method of Bligh and Dyer^41^, dried using in a centrifugal evaporator (Sakuma Seisakusho, Tokyo, Japan), and reconstituted in methanol. Products were quantified by LC-MS. There were separated on an ACQUITY UPLC BEH C8 column (1.7 µm, 2.1 × 30 mm, Waters, Milford, MA) using a linear gradient of solvent B (acetonitrile; Wako) over solvent A (20 mM NH_4_HCO_3_/water; Wako) by an ACQUITY ultra performance liquid chromatography system (Waters, Milford, MA). Selected reaction monitoring was performed with a TSQ Vantage triple stage quadrupole mass spectrometer (Thermo Fisher Scientific Life Sciences) for determination of assay products.

### siRNA transfection

ATDC5 cells were seeded in 6-well tissue culture plates at a density 3.0 × 10^5^ cells/well and cultured in α-MEM without antibiotics for 24 h. The cells were transfected with 100 nM siRNAs using Lipofectamine RNAiMAX (Thermo Fisher Scientific Life Sciences) according to the manufacturer’s instructions. Control siRNA (Santa Cruz Biotechnology; SC: 37007), used as a negative control, consisted of a scrambled sequence that would not affect any cellular message and function. Cells were incubated for another 24 h. Cells were further cultured in α-MEM with 50 ng/mL ascorbic acid and 1% ITS to induce chondrogenic differentiation before subsequent experiments.

### Trypan blue exclusion test

Cell viability was valued by trypan blue dye exclusion. ATDC5 cells were plated onto 24-well culture plates at a density 6.0 × 10^4^ cells/well. On the next day, the cells were transfected with control siRNA or LPCAT4 siRNA. At 24 h after transfection, cells were stained with trypan blue stain solution (Nacalai Tesque), and stained cells were counted with a hemocytometer.

### WST-8 assay

Cell viability was additionally evaluated by WST-8 assay. ATDC5 cells were seeded in 24-well culture plates at a density 6.0 × 10^4^ cells/well. On the next day, the cells were transfected with control siRNA or LPCAT4 siRNA. The WST-8 assay was conducted with a Cell Counting kit-8 (Dojindo, Kumamoto, Japan), according to the manufacturer’s instructions. At 24 h after transfection, the cells were incubated with 10% WST-8 reagent for 4 h at 37 °C. One hundred microliters of supernatants was transferred from each well to a fresh 96-well plate. Cell viability was determined by measuring the absorbance at 450 nm with a Multiskan JX.

### ALP activity assay

ATDC5 cells were seeded in 24-well culture plates at a density 6.0 × 10^4^ cells/well. On the next day, the cells were transfected with control siRNA or LPCAT4 siRNA for 24 h. The cells were then cultured in α-MEM with 50 ng/mL ascorbic acid and 1% ITS for 15 days. For measurement of ALP activity, ATDC5 cells were suspended in 600 μL Cell Lysis Buffer. ALP activity was measured with Lab Assay ALP (Wako), according to the manufacturer’s instructions, and normalized to the protein concentration, which was measured by DC protein assay kit using BSA as a standard.

### Statistics analyses

All statistical calculations were done using Microsoft Excel (Microsoft, Redmond, WA, USA) or SPSS 15.0 software (IBM, Armonk, NY, USA). Data were expressed as the mean ± SD and analyzed by one-way or two-way analysis of variance (ANOVA), followed by a post-hoc test (Bonferroni’s correction) for multiple comparisons. Differences between two groups were analyzed by two-tailed unpaired Student’s *t*-test. *p* < 0.05 was considered statistically significant.

## Electronic supplementary material

Supplementary Information
