# Supplementary material for: Lysophosphatidylcholine acyltransferase 4 is involved in chondrogenic differentiation of ATDC5 cells
Source: Sci Rep. 2017 Dec 1;7:16701. doi: 10.1038/s41598-017-16902-4 (PMC5711957; doi:10.1038/s41598-017-16902-4)
Supplement: Supplementary file 1 — Supplementary Information [file 41598_2017_16902_MOESM1_ESM.pdf]

Lysophosphatidylcholine acyltransferase 4 is involved in chondrogenic differentiation of ATDC5 cells

Shirou Tabe[1,2], Hisako Hikiji[3*], Wataru Ariyoshi[1], Tomomi Hashidate-Yoshida[4], Hideo Shindou[4,5], Takao Shimizu[4,6], Toshinori Okinaga[1], Yuji Seta[7], Kazuhiro Tominaga[2], and Tatsuji Nishihara[1]

[1]Division of Infections and Molecular Biology, Department of Health Promotion, Kyushu Dental University, Kitakyushu, Fukuoka 803-8580, Japan

[2]Division of Oral and Maxillofacial Surgery, Department of Science of Physical Functions, Kyushu Dental University, Kitakyushu, Fukuoka 803-8580, Japan

[3]School of Oral Health Sciences, Kyushu Dental University, Kitakyushu, Fukuoka 803-8580, Japan

[4]Department of Lipid Signaling, Research Institute, National Center for Global Health and Medicine, Shinjuku-ku, Tokyo 162-8655, Japan

[5]Agency for Medical Research and Development-Core Research for Evolutional Medical Science and Technology (AMED-CREST), AMED, Chiyoda-ku, Tokyo 100-0004, Japan

[6]Department of Lipidomics, Graduate School of Medicine, The University of Tokyo, Bunkyo-ku, Tokyo 113-0033, Japan

[7]Division of Anatomy, Department of Health Improvement, Kyushu Dental University, Kitakyushu, Fukuoka 803-8580, Japan

*Corresponding author: r09hikiji@fa.kyu-dent.ac.jp.

Supplementary information

Supplemental TABLE 1.

Phospholipid composition of phosphatidylcholine (PC) during chondrogenic differentiation of ATDC5 cells.

**PC species**	**day 0 (%)**	**day 5 (%)**	**day 10 (%)**	**day 15 (%)**
PC30:0	0.95 ± 0.01	1.20 ± 0.01	1.17 ± 0.05	1.18 ± 0.11
PC30:1	1.70 ± 0.03	2.40 ± 0.26	2.82 ± 0.11	2.10 ± 0.05
PC32:0	6.40 ± 0.25	4.89 ± 0.12	5.48 ± 0.05	6.95 ± 0.26
PC32:1	8.37 ± 0.19	8.81 ± 0.08	9.70 ± 0.25	9.12 ± 0.27
PC32:2	0.82 ± 0.03	0.98 ± 0.06	1.25 ± 0.03	1.12 ± 0.05
PC34:0 or oPC36:7 or ePC36:6	3.91 ± 0.10	3.34 ± 0.08	3.10 ± 0.10	3.30 ± 0.09
PC34:1	32.08 ± 0.41	30.36 ± 1.30	27.31 ± 0.83	29.60 ± 0.69
PC34:2	5.71 ± 0.14	6.35 ± 0.23	6.28 ± 0.24	6.65 ± 0.28
PC36:0 or oPC38:7 or ePC38:1	0.98 ± 0.05	1.10 ± 0.04	1.13 ± 0.03	0.98 ± 0.06
PC36:1	10.77 ± 0.22	13.09 ± 0.51	12.82 ± 0.16	11.42 ± 0.27
PC36:2	16.08 ± 0.17	17.89 ± 0.59	15.61 ± 0.30	14.35 ± 0.33
PC36:3	2.35 ± 0.09	1.60 ± 0.05	2.33 ± 0.04	2.57 ± 0.10
PC36:4	0.62 ± 0.02	0.31 ± 0.01	0.66 ± 0.02	0.93 ± 0.05
PC38:1 or oPC40:8 or ePC40:7	0.99 ± 0.04	1.05 ± 0.05	1.06 ± 0.02	0.78 ± 0.02
PC38:2 or oPC40:9 or ePC40:8	2.85 ± 0.04	3.65 ± 0.13	4.22 ± 0.09	3.38 ± 0.07
PC38:3 or oPC40:10 or ePC40:9	2.28 ± 0.10	1.65 ± 0.10	2.62 ± 0.07	2.64 ± 0.08
PC38:4 or oPC40:11 or ePC40:10	1.74 ± 0.01	0.97 ± 0.02	1.79 ± 0.05	2.08 ± 0.03
PC38:5	0.68 ± 0.04	0.34 ± 0.01	0.64 ± 0.04	0.85 ± 0.02

The signal intensities for each species were summed, and the percentage of each species was calculated. The data show mean ± S.D., n = 3. Compared with day 0, post hoc test (Bonferroni's test) after two-way analysis of variance. A significant difference was not recognized between all PC species.

Supplemental Figure 1.

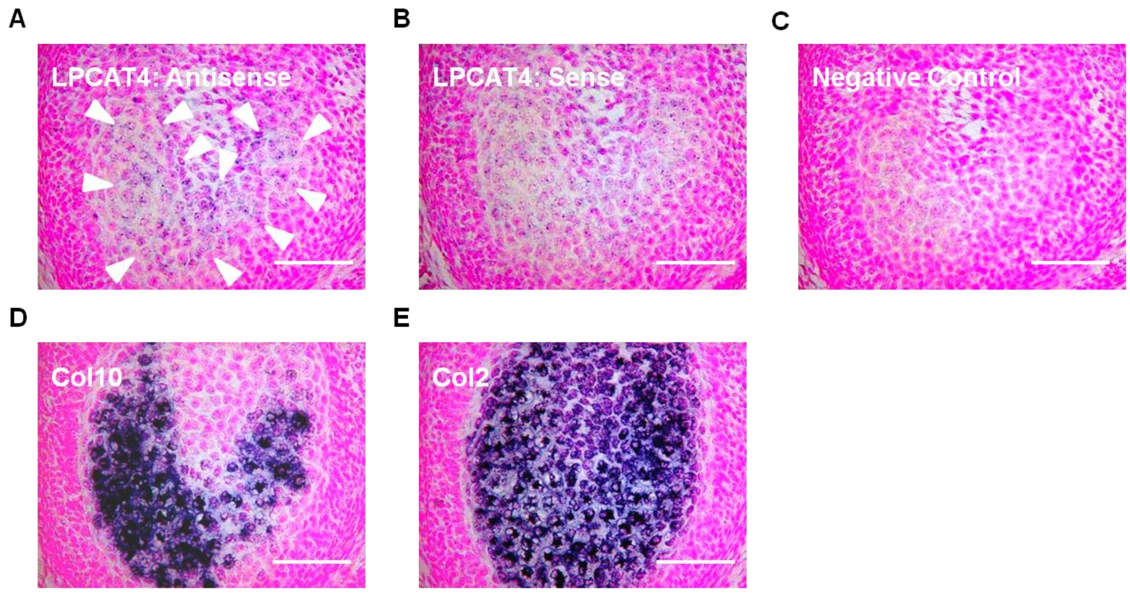

Supplemental Figure 1. LPCAT4 mRNA expression within the hypertrophic zone of cartilage. First rib cartilages were dissected from embryos at E18.5 and were used for *in situ* hybridization. (A) The antisense probe produced a stronger signal in the hypertrophic zone of cartilage (white arrowheads) than (B) the sense probe. (C) Counter-staining with Kernechtrot staining solution as a negative control. (D) Col10 mRNA is expressed within the hypertrophic cartilage zone of cartilage. (E) Col2 mRNA is expressed within the prehypertrophic and hypertrophic zones of cartilage. Scale bar, 100 μm.

Supplemental TABLE 2.

Phospholipid composition of phosphatidylcholine (PC) in ATDC5 cells transfected with control (Ctr) or lysophosphatidylcholine acyltransferase 4 (LPCAT4)

**PC species**	**Ctr si (%)**	**LPCAT4 si (%)**
PC30:0	0.96 ± 0.03	1.04 ± 0.01
PC30:1	2.23 ± 0.18	2.14 ± 0.05
PC32:0	6.35 ± 0.55	6.45 ± 0.22
PC32:1	8.73 ± 0.46	9.21 ± 0.18
PC32:2	1.15 ± 0.04	1.38 ± 0.03
PC34:0 or oPC36:7 or ePC36:6	3.33 ± 0.12	3.23 ± 0.07
PC34:1	28.39 ± 0.77	27.92 ± 0.82
PC34:2	6.58 ± 0.39	6.82 ± 0.26
PC36:0 or oPC38:7 or ePC38:1	1.02 ± 0.01	0.98 ± 0.05
PC36:1	11.37 ± 0.30	10.90 ± 0.04
PC36:2	13.91 ± 0.20	13.58 ± 0.15
PC36:3	3.28 ± 0.20	3.44 ± 0.05
PC36:4	1.29 ± 0.08	1.32 ± 0.06
PC38:1 or oPC40:8 or ePC40:7	0.81 ± 0.02	0.78 ± 0.01
PC38:2 or oPC40:9 or ePC40:8	3.43 ± 0.07	3.48 ± 0.01
PC38:3 or oPC40:10 or ePC40:9	3.34 ± 0.04	3.47 ± 0.02
PC38:4 or oPC40:11 or ePC40:10	2.79 ± 0.12	2.80 ± 0.15
PC38:5	1.06 ± 0.02	1.07 ± 0.07

The signal intensities for each species were summed, and the percentage of each species was calculated. The data show mean ± S.D., n = 3. Compared with cells transfected with control siRNA, post hoc test (Bonferroni's test) after two-way analysis of variance. A significant difference was not recognized between all PC species.

Supplementary materials and methods

Measurement of PC

Lipids were extracted by the method of Bligh and Dyer [1] from proteins (1 μg) used for analysis of LPCAT enzymatic activity. PC was measured by liquid chromatography-selected reaction monitoring/mass spectrometry (LC-SRM-MS) as previously described[2, 3]. The LC system was a Nexera UHPLC system and the MS system was a triple quadrupole mass spectrometer, LCMS-8050 (Shimadzu Corporation, Kyoto, Japan). An Acquity UPLC BEH C8 column (1.7 μm, 2.1 mm × 100 mm, Waters) was used for separation of PC, and column temperature was 47°C. Solvent A was 5 mM ammonium bicarbonate (Wako, Osaka, Japan), solvent B was acetonitrile (Wako) and solvent C was isopropanol (Wako). The LC solvent gradient was as follows: 0 min (solvent A = 75%/solvent B = 20%/solvent C = 5%)–20 min (20/75/5)–40 min (20/5/75)–45 min (5/5/90)–50 min (5/5/90)–55 min (75/20/5). Flow rate was 0.35 ml/min and 5 μl of sample was applied. PC was identified by the precursor ion of m/z=184.

In situ hybridization

C57BL/6N mice were purchased from Charles River (Yokohama, Japan). The animals

used in this study were maintained and handled according to protocols approved by Kyushu Dental University Animal Care. The day of vaginal plug detection was considered to be embryonic day 0.5 (E0.5). Pregnant mice were sacrificed on E18.5 by administering an overdose of sevoflurane (Pfizer, Tokyo, Japan). The embryos were surgically removed, fixed with G-Fix (Genostaff, Tokyo, Japan), and embedded in paraffin on CT-Pro20 (Genostaff) using G-Nox (Genostaff) as a less toxic organic solvent for xylene, and sectioned at 8 μm. *In situ* hybridization was carried out using the ISH Reagent Kit (Genostaff) according to the manufacturer's instructions. Tissue sections were deparaffinized with G-Nox and rehydrated through an ethanol series and phosphate buffered saline (PBS). The sections were fixed with 10% neutral buffered formalin (NBF) for 15 min at room temperature (RT) and treated with 4 μg/ml proteinase K (Wako) in PBS for 10 min at 37°C. After washing with PBS, the sections were refixed with 10% NBF for 15 min at RT, placed in 0.2 N HCl for 10 min at RT, washed in PBS, and placed in a coplin jar containing 1× G-Wash (Genostaff) and equal to 1× saline sodium citrate. Hybridization was performed with probes at concentrations of 300 ng/ml in G-Hybo-L (Genostaff) for 16 h at 60 °C. After hybridization, the sections were washed in 1× G-Wash for 10 min at 60 °C and 50% formamide in 1× G-Wash for 10 min at 60 °C. Then, the sections were washed twice in 1× G-Wash for

10 min at 60 °C, twice in 0.1× G-Wash for 10 min at 60 °C, and twice in Tris-buffered saline containing 0.1% Tween-20 (TBST) at RT. After treatment with 1× G-Block (Genostaff) for 15 min at RT, the sections were incubated with anti-digoxigenin alkaline phosphatase conjugate (Roche, Tokyo, Japan) diluted 1:2000 with 50× G-Block in TBST for 1 h at RT. The sections were washed twice in TBST and then incubated in 100 mM NaCl, 50 mM $MgCl_2$, 0.1% Tween20, and 100 mM Tris-HCl (pH 9.5). The sections were stained overnight in NBT/BCIP solution (Sigma-Aldrich, St. Louis, MO, USA) and then washed in PBS. The sections were counterstained with Kernechtrot staining solution (Muto Chemical, Tokyo, Japan) and mounted with G-Mount (Genostaff). The LPCAT4 probe sequence used for the assay corresponded to the complementary sequence of LPCAT4 nucleotides 1384–2246 (GenBank accession number NM_026037.3). Col2 (MP-C-021) and Col10 (MP-C-024) probes were purchased from Genostaff.

Supplementary references

1. Bligh, E.G. & Dyer, W.J. A rapid method of total lipid extraction and purification. *Can. J. Biochem. Physiol.* **37,** 911–917 (1959).
2. Hashidate-Yoshida, T. et al. Fatty acid remodeling by LPCAT3 enriches

arachidonate in phospholipid membranes and regulates triglyceride transport. *ELife*. **4,** e06328 (2015).

3. Harayama, T. et al. Lysophospholipid acyltransferases mediate phosphatidylcholine diversification to achieve physical properties in vivo. *Cell Metabolism.* **20,** 295-305 (2014).
